# Diferencia de Riesgos, Riesgo Relativo y Odds Ratio

**DOI:** 10.31083/RN33481

**Published:** 2025-03-07

**Authors:** Carmen Carazo-Díaz, Luis Prieto-Valiente

**Affiliations:** ^1^Facultad de Medicina. (UCAM) Universidad Católica de Murcia, E-30107 Murcia, España; ^2^Sociedad Científica de Investigación Biomédica (SCIB), 07010 Palma de Mallorca, España

**Keywords:** frecuencia relativa, riesgo relativo, odds ratio, caso-control, epidemiología pública, relative frequency, relative risk, odds ratio, case-control, public epidemiology

## Abstract

Típicamente la frecuencia relativa de una enfermedad se expresa con la proporción de afectados, que también se puede expresar como porcentaje, tanto por mil... Menos usado, pero de utilidad en muchos casos, es la Odd que es el número de enfermos por cada sano o, lo que es lo mismo, la proporción de enfermos partido entre la proporción de sanos. Para evaluar el efecto pernicioso o positivo de un factor en relación con una enfermedad se compara la proporción de enfermos cuando está presente el factor con la proporción cuando no lo está. La no igualdad de dos proporciones puede expresarse de muchos modos, de los cuales los más frecuentemente usados son: la diferencia de proporciones o de porcentajes, denominada Diferencia de Riesgos, DF, el cociente de proporciones, denominado Riesgo Relativo, RR y el cociente de las Odds, denominado Odds Ratio, OR. Con enfermedades de baja frecuencia, la OR es muy próxima al RR lo que resulta de gran utilidad en los estudios denominados “Caso-Control”.

## Abstract (en)

In epidemiology, the relative frequency of a disease is expressed as the proportion of individuals affected, typically expressed as a percentage, or per thousand individuals. Another important measure is the odds, which represents the ratio of affected individuals to unaffected individuals, calculated by dividing by the proportion of affected individuals by the proportion of unaffected individuals. To assess whether a specific factor increases or decreases the risk of disease, researchers compare the proportion of affected individuals in an exposed group (where the factor is present) with an unexposed group (where the factor is absent). This comparison can be quantified using three key measures: Risk Difference (RD): The absolute difference in disease risk between the exposed and unexposed groups. Relative Risk (RR): The ratio of disease risk in the exposed group to that in the unexposed group. Odds Ratio (OR): The ratio of the odds of disease in the exposed group to the odds in the unexposed group. While risk reflects the proportion of individuals affected within a population, odds represent the ratio of affected to unaffected individuals. The OR is particularly useful in case-control studies because it can approximate the RR when diseases are rare, providing valuable insights even when direct risk calculations are not feasible.

## 1. Introducción 

Para comparar la frecuencia de una característica en dos grupos distintos lo intuitivo sería contar el número de individuos con la característica en cada grupo, es decir calcular la FRECUENCIA ABSOLUTA en cada uno. Sin embargo comparar solamente frecuencias absolutas puede inducir a error.

Por ejemplo, si se quiere saber si en las personas con la variante genética “K^+^” aumenta la ocurrencia de asma, se cuenta el número de asmáticos en el grupo con la mutación, “K^+^”, y en el grupo que no la tiene, “K^–^”. Si se encuentra que en el grupo “K^–^” son asmáticos 16000 y en el grupo “K^+^” son asmáticos 4000, ello nos haría pensar, en un primer momento, que tener la mutación disminuye el riesgo de tener asma, lo que podría no ser cierto.

Para comparar cuan frecuente es una característica en dos grupos hay que tener en cuenta la cantidad total de individuos en cada grupo y usar la FRECUENCIA RELATIVA, FR, que relaciona el tamaño de una parte con el tamaño total de un colectivo. Se puede expresar como “*proporción*” o “*porcentaje*” o “*tanto por mil*” o “*tanto por cien mil*” o “*tanto por millón*”…, si la proporción es muy pequeña, con varios ceros tras el punto decimal.

Si por ejemplo, son 16000 asmáticos entre 4000000 “K^–^”, la FR de asmáticos puede expresarse como proporción 16/4000 = 0.004 o porcentaje = proporción × 100 = 0.004 × 100 = 0.4% o, de manera más operativa, como tanto por mil = proporción × 1000 = 0.004 × 1000 = 4, es decir, son asmáticos 4 de cada mil “K^–^”.

Y si son 4000 con asma entre 200000 “K^+^”, la FR de asmáticos se puede expresar como proporción 4000/200000 = 4/200 = 0.02 o como porcentaje = 0.02 × 100 = 2%, es decir, 2 de cada cien o como tanto por mil = 0.02 × 1000 = 20. Son asmáticos 20 de cada mil “K^+^”. Es decir, la mutación resulta ser factor de riesgo.

A la Frecuencia Relativa, FR, de individuos “K^–^” con asma se le llama RIESGO ESPECIFICO de los no expuestos, R_0_ = 0.004. Y a la Frecuencia Relativa, FR, de individuos “K^+^” con asma se le llama RIESGO ESPECIFICO de los expuestos, R_1_ = 0.02.

## 2. Diferencias de Riesgos y Riesgo Relativo

Siguiendo con el ejemplo, en el grupo “K^–^” son asmáticos 4 por mil y en el grupo “K^+^” son asmáticos 20 por mil. Expresado en proporciones decimos que la diferencia es 0.020 – 0.004 = 0.016, o 16 por mil, es decir, si comparamos dos colectivos, uno de 1000 personas sin la variante y otro de mil personas con la variante, el segundo tiene 16 casos más de asma.

La no igualdad de riesgos también se puede expresar como cociente. El cociente de estas dos cantidades, 0.020/0.004 = 20/4 = 5 se denomina RIESGO RELATIVO, RR. El riesgo de ser asmático es 5 veces mayor en el grupo con mutación.

Veamos un segundo ejemplo en el que factor es beneficioso, es decir, disminuye la proporción de enfermos. En un colectivo de 2000 sedentarios 1600 sufren gripe invernal, R_0_ = 0.80 u 80% y en un colectivo de 3000 deportistas sufren gripe invernal 1800, R_1_ = 0.60 ó 60%. En este ejemplo el ejercicio baja la frecuencia relativa en 20 puntos porcentuales. El Riesgo Relativo es RR = 0.6/0.8 = 0.75. Decimos “Hacer ejercicio reduce el riesgo de tener gripe al 75%”, o también “Hacer ejercicio reduce el riesgo de tener gripe en un 25%” [1, 2].

La igualdad de riesgos se puede expresar diciendo que su diferencia es cero o que su cociente es uno.

## 3. Concepto de “Odd” y “Odds Ratio” 

Otro modo de indicar la cantidad relativa de individuos con cierta enfermedad, digamos E, en un colectivo es reportar cuantos tienen esa característica por cada uno que no la tiene. A esa cantidad se la llama Odd. Se obtiene dividiendo el número de individuos que tiene E por el número de individuos sin E. Y se obtiene el mismo resultado dividiendo la proporción de los que tiene E por la proporción de los que no la tienen. Si llamamos P a la proporción de individuos, la Odd es P/(1 – P).

Ejemplos:

- Si en un grupo de 20 personas son enfermas, E, 15 de ellas, la FR de E es 15/20 = 0.75, la Odd es 15/5 = 3 y también 0.75/0.25 = 3 y dice que hay 3 E por cada no E.

- Si en un grupo de 20 personas hay 10 médicos, FR de médicos es 0.5, la Odd es 1, es decir, hay un médico por cada no médico.

- Si entre la 200 personas hay 40 rubios, la FR de rubios es 40/200 = 0.2, la Odd es 40/160 = 0.25, o también 0.2/0.8 = 1/4 = 0.25, es decir, hay 0.25 rubios por cada no rubio. Ciertamente, NO tiene sentido hablar de “0.25 personas”, pero ese valor toma sentido si multiplicamos por 4 y decimos que hay 1 rubio por cada 4 no rubios, o multiplicamos por 100 y decimos que hay 25 rubios por cada 100 no rubios.

Así como el RR se define como el cociente de dos Riesgos Específicos, la ODDS RATIO, OR, se define como el cociente de dos Odd´s: la Odd de enfermos en los expuestos al factor dividido por la Odd de enfermos en los no expuestos. La igualdad de riesgos se puede expresar diciendo que el cociente de sus Odds, es decir la OR, es uno. La OR juega papel importante en la regresión logística, técnica avanzada de análisis estadístico muy usada en la investigación médica [2, 3].

Veamos como ejemplo una población donde de un total de 300000 individuos fuman 100000, están enfermos (“D^+^”) 50000 y son enfermos y fumadores 40000.

**Table:** 

	Fuman	No Fuman	TOTAL	
D^+^	A = 40000	B = 10000	M_1_ = 50000	P1 = 4/5 = 0.80
D^–^	C = 60000	D = 190000	M_0_ = 250000	P_0⁢ _ = 6/25 = 0.24
Total	N_1_ = 100000	N_0_ = 200000	N = 300000	P_1_/P_0_ = 0.80/0.24 = 3.33
	R_1_ = 0.40	R_0_ = 0.05	RR = 0.40/0.05 = 8	

El Riesgo Especifico de enfermar en fumadores es R_1_= 40000/100000 = 0.40, el Riesgo Especifico de enfermedad en no fumadores es R_0_= 10000/200000 = 0.05 y el Riesgo Relativo del fumar para esa enfermedad es RR = 0.40/0.05 = 8, es decir, la proporción de enfermos es 8 veces mayor en fumadores.

A partir de los Riesgos Específicos podemos calcular las Odds y la OR. La Odd de enfermar en fumadores es Odd (R_1_) = 0.40/0.60 = 40000/60000 = 0.6667, la Odd de enfermar en no fumadores es Odd (R_0_) = 0.05/0.95 = 10000/190000 = 0.0526 y la Odds Ratio es OR = Odd (R_1_)/Odd (R_0_) = 0.6667/0.0526 = 12.67.

También serán útiles las siguientes cantidades: proporción de enfermos que fuman, P_1_ = 40000/50000 = 0.80, proporción de sanos que fuman, P_0_= 60000/250000 = 0.24 y el cociente P_1_/P_0_ = 0.80/0.24 = 3.33, es decir, la proporción de fumadores es 3.33 veces mayor en enfermos que en sanos. Estas proporciones son las que se estiman en los diseños tipo “Caso-Control”, como veremos en la siguiente nota estadística [4].

## 4. Coincidencia en Población de or (R´S) y or (P´S)

Siguiendo con el ejemplo del apartado anterior, en la población el cociente de los Riesgos Específicos, RR = R_1_/R_0_ = 8, no tiene el mismo valor que el cociente P_1_/P_0_ = 0.80/0.24 = 3.33.

Pero si calculamos las Odd´s con P_1_ y P_0_, se tiene que la Odds de fumar en enfermos es Odd (P_1_) = 0.80/0.20 = 4, la Odds de fumar en sanos es Odd (P_0_) = 0.24/0.76 = 0.3158 y el cociente de estas dos Odd´s es OR (P´s) = 4/0.3158 = 12.67.

Ocurre siempre que el cociente de las Odd´s toma igual valor cuando se calcula con las P´s (FR de fumadores en enfermos y en sanos) que cuando se calcula con las R´s (FR enfermos en fumadores y en no fumadores). Si se conocen las cantidades A, B, C y D del ejemplo anterior, el modo más cómodo de calcular la OR es como cociente de productos cruzados:

OR=A⋅D/B⋅C=400×1900/100×600=12.67

Como ejercicio útil, el lector puede inventar los datos para una población cualquiera y comprobar que la OR calculada con las P´s toma igual valor que la OR calculada con las R´s. Esta igualdad es de gran utilidad en los estudios llamados “Caso-Control” y lo comentaremos próximamente en una nota estadística.

## 5. Proximidad Entre los Valores de Odds Ratio y Riesgo Relativo 

Una vez comprobado que son iguales las dos OR´s veamos que este valor único de OR se aproxima mucho al valor del RR si los Riesgos Específicos toman valores próximos a cero.

Sea la siguiente población, con exposición “E^+^” y enfermedad “D^+^”:

**Table:** 

	E^+^	E^–^	Total	
D^+^	1000	200	1200	P_1_ = 10/12 = 0.833
D^–^	99000	199800	298800	P_0_ = 99/2988 = 0.033
Total	100000	200000	300000	
	R_1_ = 0.010	R_0_ = 0.001	RR = 10	

La Odd de enfermar en expuestos es Odd (R_1_) = 0.0101, la Odd de enfermar en no expuestos es Odd (R_0_) = 0.0010 y la Odds Ratio es OR = 10.1, que, como sabemos, coincide con la calculada a partir de las P_1_ y P_0_ y con la calculada como cociente de productos cruzados, y es un valor muy próximo al RR = 10.

Esta similitud entre el RR y la OR ocurre siempre que los Riesgos Específicos son próximos a cero, digamos si las R‘s son menores de 0.10, y es más acusada cuánto más pequeños son dichos riesgos. Esta proximidad también es de gran utilidad en los estudios llamados “Caso-Control”, como veremos en otra próxima nota estadística.

## 6. Conclusión

Para evaluar el efecto pernicioso o positivo de un factor en relación con una enfermedad se compara la proporción de enfermos, Riesgo Especifico de enfermar, cuando está presente el factor con la proporción de enfermos cuando no está presente.

La no igualdad de dos Riesgos Específicos o proporciones puede expresarse de muchos modos. Los más frecuentes son como Diferencia de Riesgos, como cociente de Riesgos, RR y con la Odds Ratio, OR. Ningún modo es más correcto que otro. Lo importante es que al usarlos el médico tenga claro lo que indica cada uno.

La OR poblacional calculada con los Riesgos Específicos de enfermar, es decir con las “R´s”, coindice con la OR poblacional calculada con la probabilidad de estar expuesto al factor, es decir con las “P´s”. Y con enfermedades de baja frecuencia, la OR es muy próxima al RR. Ambas circunstancias son de gran utilidad en los estudios denominados “Caso-Control”.

## Disponibilidad de Datos y Materiales

Los datos contenidos en el manuscrito son ficticios con finalidad docente.

## Contribuciones de los Autores

CC se encargó de la redacción principal del texto. LP colaboró aportando los ejemplos aclaratorios y realizando la búsqueda de bibliografía complementaria. Dos autores han contribuido en la preparación del manuscrito y en los cambios editoriales. Dos autores han leído y aprobado la versión final del manuscrito. Dos autores han participado plenamente en este trabajo y están de acuerdo en asumir la responsabilidad de todos los aspectos de este trabajo.

## Aprobación Ética y Consentimiento Informado

No aplicable.

## Agradecimientos

No aplicable.

## Financiación

Esta investigación no recibió financiación externa.

## Conflicto de Intereses

Los autores declaran no tener conflictos de interés.
